# Exploring the Frequency and Risk Factors of Hyperprogressive Disease in Patients with Advanced Melanoma Treated with Immune Checkpoint Inhibitors

**DOI:** 10.3390/curroncol31100472

**Published:** 2024-10-18

**Authors:** Caner Acar, Haydar Çağatay Yüksel, Gökhan Şahin, Fatma Pinar Açar, Burçak Karaca

**Affiliations:** Division of Medical Oncology, Departmant of Internal Medicine, Ege University Medical Faculty, 35100 Izmir, Turkey; haydar.cagatay.yuksel@ege.edu.tr (H.Ç.Y.); gokhan.sahin@ege.edu.tr (G.Ş.); fatma.pinar.acar@ege.edu.tr (F.P.A.); burcak.karaca@ege.edu.tr (B.K.)

**Keywords:** hyperprogressive disease, immune checkpoint inhibitors, advanced melanoma, risk factors, overall survival

## Abstract

Hyperprogressive disease (HPD) is described as the unexpected rapid growth of a tumour accompanied by a decline in performance status. While immune checkpoint inhibitors (ICIs) have improved outcomes in advanced melanoma, HPD remains a significant challenge in a subset of patients. Although HPD has been extensively studied in various solid tumours, research specifically focusing on advanced melanoma remains limited. We analysed 158 advanced melanoma patients, with 66.5% (*n* = 105) receiving anti-PD-1 and 33.5% (*n* = 53) receiving nivolumab plus ipilimumab. The median overall survival was 4.9 months for patients with HPD compared to 8.9 months for those with progressive disease without HPD (*p* = 0.014). Factors associated with HPD included liver metastasis (*p* = 0.002), three or more metastatic sites (*p* < 0.001), elevated lactate dehydrogenase levels (*p* = 0.004), and Eastern cooperative oncology group performance status ≥2 (*p* = 0.023). Multivariate analysis identified the Royal Marsden Hospital score (HR 3.675, 95% CI: 1.166–11.580, *p* = 0.026) as an independent risk factor for HPD, with the MDA-ICI score also trending towards significance (HR 4.466, 95% CI: 0.947–21.061, *p* = 0.059). This study provides valuable insights into the frequency and factors associated with HPD in advanced melanoma patients treated with ICIs, highlighting the relevance of clinical markers and scoring systems in predicting HPD risk.

## 1. Introduction

The treatment of advanced melanoma has been revolutionised with the use of immune checkpoint inhibitors (ICIs). These agents are notable for their ability to induce durable responses and relatively favourable tolerability profiles [1]. However, with the increasing use of ICIs, atypical response patterns, such as pseudoprogression, mixed responses, and hyperprogressive disease (HPD), have been recognised.

Although there is no consensus on its precise definition, HPD is generally described as the unexpected rapid growth of a tumour accompanied by a decline in performance status. Meta-analyses have reported that the frequency of HPD after ICI therapy in solid tumours ranges from 5.9% to 43.1% [2]. Similarly, studies focusing on advanced melanoma have shown highly variable frequencies ranging from 1.3% to 43% [3,4].

HPD was first described by Champiat et al. in 2017, and it is characterised by disease progression at first evaluation and a two-fold or greater increase in tumour growth rate (TGR) shortly after treatment initiation [5]. However, because these definitions require both pre-baseline and post-baseline imaging, they are considered impractical for routine clinical use. To address these challenges, clinical criteria such as time-to-treatment failure (TTF) and deterioration in Eastern cooperative oncology group (ECOG) performance status, as well as alternative radiologic criteria like an increase in tumour burden, have been proposed as more accessible methods for HPD diagnosis [6].

In addition to its definition and diagnostic criteria, there is ongoing debate about whether HPD is caused by ICI therapy or is a consequence of the aggressive nature of the disease itself [7]. Studies have suggested that HPD is more frequently observed following ICI therapy compared to chemotherapy or targeted agents [8].

The tumour microenvironment plays a crucial role in the response to ICI therapy and is considered a potential mechanism underlying HPD. Possible mechanisms following ICI therapy include an increase in PD-1^+^ regulatory T cells, an increase in immunosuppressive cytokines, and an increase in M2 macrophages [6,9,10]. Additionally, certain genomic alterations, such as MDM2/MDM4 amplification and EGFR mutations, have been implicated in HPD [11].

Regardless of the underlying cause, previous studies have shown that patients experiencing HPD have poorer overall survival (OS) and progression-free survival compared to those with other progressive disease (PD) [8]. Therefore, there is growing interest in identifying HPD risk factors to optimise treatment strategies. Previous studies examining HPD have primarily focused on non-melanoma solid tumours, with limited research available on melanoma. Given the complexity of the immune response during ICI treatment, systemic inflammatory and nutritional indices have been explored as potential markers for predicting OS in patients with solid tumours treated with ICI [12,13,14]. Furthermore, well-established scoring systems such as the Royal Marsden Hospital (RMH) score, the MD Anderson Immune Checkpoint Inhibitor (MDA-ICI) score, and the Gustave Roussy Immune Score (GRIm score) have been used to stratify patients based on their likelihood of rapid disease progression, making them invaluable tools in evaluating HPD risk [15,16]. This study aimed to evaluate the frequency of HPD and its associated risk factors in patients with advanced melanoma receiving anti-PD-1 monotherapy or anti-PD-1 combined with anti-CTLA-4 therapy.

## 2. Materials and Methods

This is a single-centre, retrospective study that included patients diagnosed with advanced melanoma who were treated with ICI and had at least one follow-up radiologic evaluation at the Department of Medical Oncology, Faculty of Medicine, Ege University, between January 2015 and November 2023. Electronic medical records were used for data subtraction. The study adhered to good clinical practice guidelines and the principles of the Declaration of Helsinki. The Institutional Ethical Review Board of Ege University Hospital approved the study.

Patients who met the following inclusion criteria were enrolled in the study: (1) aged 18 years or older, (2) having histologically proven unresectable stage III or IV (advanced) melanoma, (3) treated with either anti-PD-1 therapy or a combination of anti-PD-1 and CTLA-4 therapy, and (4) having a baseline and at least one follow-up radiological evaluation. Patients with prior anti-PD-1 therapy who exhibited resistance were excluded from the study.

HPD diagnosis was made in patients who were identified as having PD according to RECISTv1.1 at the first evaluation and met at least three of the following criteria: (1) TTF of less than 2 months, (2) an increase of ≥50% in the sum of target lesions between baseline and the first radiologic evaluation, (3) the appearance of at least two new lesions in an organ already involved between baseline and the first radiologic evaluation, (4) the spread of disease to a new organ between baseline and the first radiologic evaluation, and (5) clinical deterioration with a decrease in ECOG performance status by ≥2 during the first 2 months [6].

Patient demographics, disease characteristics, body mass index (BMI), previous systemic therapies, ECOG performance status, and baseline laboratory values prior to ICI therapy, including the absolute eosinophil count (AEC), lactate dehydrogenase (LDH), albumin level, neutrophil–lymphocyte ratio (NLR), platelet–lymphocyte ratio (PLR), lymphocyte–monocyte ratio (LMR), and mean platelet volume to lymphocyte ratio (MPV/lymphocyte ratio), were documented. Additionally, the pan-immune-inflammation value (PIV), Systemic Immune-Inflammation Index (SII), Systemic Inflammatory Response Index (SIRI), and haemoglobin, albumin, lymphocyte, and platelet (HALP) were calculated according to previously established methods [17,18,19,20]. RMH score, MDA-ICI score, and GRIm score were calculated based on previous studies [15,16,21]. Cutoff values for NLR and PLR were derived from previous studies [22]. Treatment failure was defined as the discontinuation of treatment due to cancer progression, drug toxicity, or death. OS was defined as the time from the first dose to death from any cause.

The statistical analyses were performed using SPSS version 26.0. After descriptive statistics were conducted, the Kolmogorov–Smirnov and Shapiro–Wilk tests were used to check whether the continuous variables followed a normal distribution. For the comparison of continuous-ordinal variables that did not follow a normal distribution between groups with and without HPD, the non-parametric Mann–Whitney U test was applied, while the Student’s T-test was used for normally distributed variables. The results were presented as median (min–max) and mean ± SD. Categorical variables between groups were compared using the Chi-square and Fisher’s exact tests, and results were expressed as numbers (percentages).

A receiver operating characteristic (ROC) analysis was conducted to determine the cutoff values for PIV, SII, SIRI, and HALP, while the median was used for the LMR and MPV/lymphocyte ratio. For parameters with statistically significant AUC values in the ROC analysis, cutoff points were calculated using sensitivity, specificity, and Youden’s index. Risk factors for HPD were evaluated using both univariate and multivariate binary logistic regression analyses. The most important independent risk factors for HPD were identified using a multivariate regression analysis of the model formed from parameters that were statistically significant in the univariate analysis. Kaplan–Meier analysis and the Log-rank test were used to compare OS times between HPD groups. The results were provided with 95% confidence intervals, and a *p*-value of <0.05 was considered statistically significant in all tests.

## 3. Results

### 3.1. Patient Characteristics

Overall, 158 patients with advanced melanoma who met the study criteria were included out of 190 patients who received anti-PD-1 and anti-PD-1-CTLA-4 inhibitor therapy between January 2015 and December 2023. HPD was observed in 24 patients (15.2%). The frequency of HPD diagnostic criteria in these patients is shown in Table S1. Most of the patients were under 65 years old (70.9%) and male (60.1%). The ECOG performance status was 0–1 in 90.5% of the patients, and the frequency of BRAF mutation was 33.5%. ICI therapy was administered as a first-line treatment in 43.7% of the patients. In 74 patients (39.0%), the best overall response was PD. Among these patients, HPD was observed in 32.4%. In terms of immune-related adverse events (irAEs), immunosuppressive therapy was required in two patients (12.5%) receiving anti-PD-1 therapy and one patient (12.5%) receiving anti-PD-1-CTLA-4 therapy in the HPD group, while in the non-HPD group, 14 patients (16.3%) on anti-PD-1 therapy and 19 patients (42.2%) on anti-PD-1-CTLA-4 therapy experienced irAEs requiring immunosuppressive treatment. No permanent treatment discontinuation due to irAEs occurred in the HPD group. However, in the non-HPD group, two patients on anti-PD-1 therapy and four patients on anti-PD-1-CTLA-4 therapy had permanent discontinuation, with one patient on anti-PD-1 therapy dying from grade 3 myocarditis.

### 3.2. ICI Type and HPD

Among the patients included in this study, 105 (66.5%) received anti-PD-1 therapy and 53 (33.5%) received a combination of anti-PD-1 and anti-CTLA-4 therapy. No difference in HPD frequency was observed between the two treatment groups (anti-PD-1 group, 15.2%; combination group, 15.1%). Among patient characteristics, patients aged 65 years or older were more common in the anti-PD-1 group (*p* = 0.044), whereas brain metastasis (*p* = 0.03), liver metastasis (*p* = 0.007), BRAF mutation (*p* = 0.012), and LDH levels >1.5 times the upper limit of normal (ULN; *p* = 0.009) were more frequent in the combination therapy group. No other significant differences were found between the clinical characteristics of the treatment groups.

### 3.3. Variables Associated with HPD

Table 1 summarises the differences between patients with and without HPD. HPD was more frequently observed in patients with liver metastasis (*p* = 0.002), those with three or more metastatic sites (*p* < 0.001), and those with an ECOG performance status ≥2. Among the laboratory parameters, HPD was more frequent in patients with LDH levels > 1.5 times the ULN (*p* = 0.004) and those with an AEC < 100/μL (*p* = 0.011). Additionally, HPD was more common in patients classified as high risk based on the RMH and MDA-ICI scores than in those in the low-risk group (*p* = 0.001 and *p* = 0.002, respectively). Table 2 provides a detailed summary of the clinical characteristics of patients with HPD. ROC analysis was performed to evaluate the predictive value of the SII, PIV, SIRI, and HALP scores for HPD, but no significant cutoff values were found (Table S2).

The results of the multivariate analysis of variables associated with HPD are presented in Table 3. For the MDA-ICI and RMH scores, multivariate analysis was performed without including the parameters that are part of these scores. Specifically, the MDA-ICI score includes ECOG performance status, LDH levels, and the presence of liver metastasis, while the RMH score includes the number of metastatic sites and LDH levels. Therefore, these parameters were excluded from the multivariate analysis to avoid collinearity. In Model 1, no significant variables were identified as independent risk factors for HPD. In Model 2, the high-risk group, according to the MDA-ICI score, showed a difference towards significance compared with the low-risk group (HR 4.466, 95% CI: 0.947–21.061, *p* = 0.059). Additionally, in this model, having three or more metastatic sites (HR 3.546, 95% CI: 1.093–11.507, *p* = 0.035) and an AEC < 100/μL (HR 2.960, 95% CI: 1.029–8.511, *p* = 0.044) were identified as independent risk factors for HPD. In Model 3, patients classified as high risk based on the RMH score (HR 3.675, 95% CI: 1.166–11.580, *p* = 0.026) and those with an ECOG performance status ≥ 2 (HR 4.523, 95% CI: 1.227–16.676, *p* = 0.023) were found to be independent risk factors for HPD.

### 3.4. Survival Data

**Figure 1:**
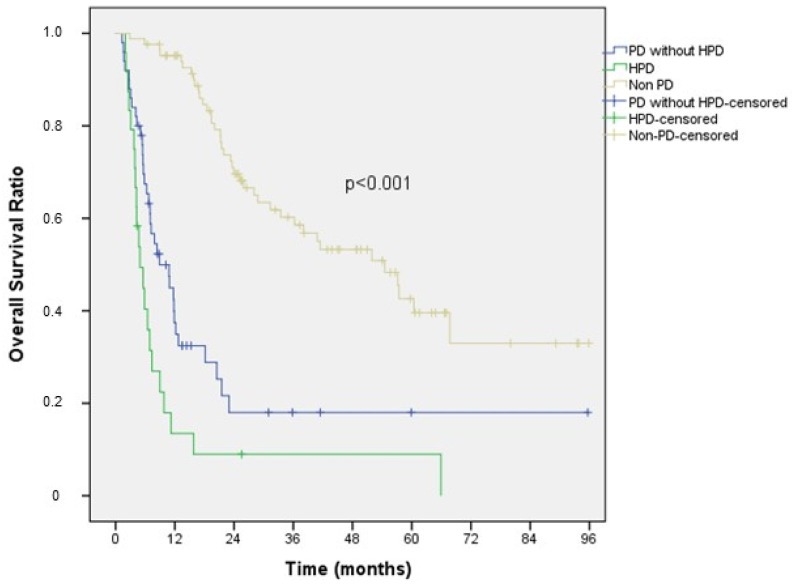
Overall survival comparison among HPD, PD without HPD, and non-PD patients.

Kaplan–Meier survival analysis of the patients is presented in Figure 1. According to our results, the median overall survival (mOS) was 4.9 months (95% CI: 3.0–6.9) in the HPD group, 8.9 months (95% CI: 4.4–13.4) in the PD without HPD group, and 54.5 months (95% CI: 37.6–71.4) in the non-PD group. The difference in mOS among the three groups was statistically significant (HPD vs. PD without HPD, *p* = 0.014; HPD vs. non-PD, *p* < 0.001).

## 4. Discussion

In our single-centre retrospective study, which reflects real-world data, the frequency of HPD in patients with advanced melanoma who received ICI therapy was 15.2%. According to a meta-analysis by Zhao et al., which included 41 studies, the frequency of HPD among patients with solid malignancies receiving ICI therapy was 13.2%. However, HPD has not been widely studied in advanced melanoma, and based on a pooled estimation from three studies, the frequency of HPD in patients with advanced melanoma was 9.9% [23].

There is no consensus on the definition of HPD, and the higher frequency observed in our study may be attributed to the definition used. Definitions that include TGR are commonly used to define HPD, but they require pre-baseline radiological imaging, making their implementation challenging in clinical practice. Additionally, these definitions do not account for the development of new lesions, which is another significant limitation [5]. In our study, we used the HPD definition proposed by Lo Russo et al. [6]. This definition offers several advantages, including ease of use in clinical practice by employing tumour burden change instead of TGR, distinguishing HPD from pseudoprogression by incorporating the ECOG performance score in the criteria, and considering the development of new lesions.

In our study, using the applied definition, patients diagnosed with HPD had a lower mOS compared to PD patients without HPD, consistent with previous studies [24]. This finding suggests that, despite ongoing debate about whether HPD is driven by the natural course of the disease or induced by ICIs, patients with HPD represent a specific subgroup of patients with PD that requires clinical recognition.

In our study, the frequency of HPD was similar between patients receiving anti-PD-1 monotherapy and those receiving a combination of anti-PD-1 and anti-CTLA-4. However, when examining the differences between the two groups, higher LDH levels and a higher frequency of liver metastases were observed in the combination group. The relationship between these variables and HPD was demonstrated in our study, and the lack of difference in HPD frequency between the groups may be due to these factors. Previous studies have shown conflicting results regarding the frequency of HPD among patients receiving combination ICI therapy. In a study recently published by Fournier et al., the frequency of HPD was 6% in patients receiving combination ICIs and 10% in those receiving anti-PD-1 therapy [25]. However, Matos et al. suggested that patients receiving combination ICIs may have a higher risk of HPD [26].

The evaluation of clinicopathological and laboratory variables associated with HPD revealed that liver metastasis, elevated LDH levels, the presence of three or more metastases, and an ECOG performance status ≥2 were associated with HPD. Previous studies assessing these factors related to HPD have predominantly included a limited number of patients with melanoma, focusing mainly on other solid tumours [24,25,27,28]. In concordance with these studies, our research demonstrated that these factors are also associated with HPD in patients with advanced melanoma. The relationship between liver metastasis and HPD may be explained by findings from previous studies on melanoma patients, which have shown that liver metastases can reduce CD8+ T-cell density at the tumour margin and exert a systemic influence on the immune system, potentially contributing to HPD development [29].

In this study, we found that an AEC < 100/μL was significantly associated with HPD development. To our knowledge, this is the first study to demonstrate the relationship between low AEC and HPD in patients with advanced melanoma. The presence of eosinophils in solid tumours has long been recognised, and their role in the response to ICIs is increasingly recognised. Recent studies have demonstrated that the interaction between innate lymphoid cells-2 and eosinophils modulates the tumour microenvironment [30,31]. Eosinophils contribute to reducing vascular leakiness, alleviating hypoxia through vessel normalisation and promoting the polarisation of macrophages towards an anti-tumorigenic M1 phenotype [32]. Additionally, increased peripheral eosinophil counts have been identified as a potential biomarker of ICI response [33]. DPP4 modulates chemokines by cleaving CCL11, reducing eosinophil infiltration and T-cell-independent anti-tumour responses [34]. Hollande et al. showed that DPP4 inhibition with ICI enhances eosinophil and T-cell anti-tumour activity, reducing the tumour burden [35]. This combination may be effective in enhancing ICI response in patients with low eosinophil counts who are at a high risk of HPD [36].

Previous studies have yielded varying results regarding the association between age and HPD. Some studies have suggested that both older and younger patients may be at risk of HPD [5,37]. In our study, patients over 65 were found to have a trend towards a lower risk for HPD, with results approaching statistical significance (*p* = 0.052). Additionally, although the NLR has been shown to be associated with HPD in some studies [28], our study did not find a significant association between NLR and HPD. Moreover, we examined the predictive significance of inflammatory indices, including PIV, SII, SIRI, and HALP scores, which have previously been shown to have predictive value for OS in patients with solid organ tumours treated with ICI. However, ROC analysis did not identify a significant cutoff value for predicting HPD. These results may reflect distinct inflammatory changes in HPD.

The Grim, MDA-ICI, and RMH scores were originally developed in Phase I trials to help select appropriate patients with a life expectancy of at least 3 months. Therefore, these scores may be useful for identifying patients at risk of early mortality and rapid disease progression [15,16,21]. In line with previous studies on solid organ tumours [24,38,39,40], our results showed that the RMH score was a significant predictor of HPD. In the multivariate analysis for the RMH score, the ECOG performance status was also identified as an independent variable. This finding suggests that the combined use of the ECOG and RMH scores could enhance the prediction of HPD. However, these results need to be validated in further studies. For the MDA-ICI score, the analysis evaluating its association with HPD showed statistical significance in the univariate analysis, whereas the multivariate analysis was close to reaching statistical significance (*p* = 0.059). While this association has been demonstrated in the literature, it has been studied less extensively than the RMH score [26,39].

This study has several limitations. As a retrospective, single-centre study, the generalisability of the findings may be limited. Additionally, there may be clinical differences between patients who received anti-PD1 monotherapy and those treated with the anti-PD1-CTLA-4 combination therapy, particularly in terms of tumour burden, prognostic factors, and treatment decisions, which could have influenced the frequency of HPD. Another limitation is the lack of TGR measurements, which restricted our ability to compare different HPD definitions.

## 5. Conclusions

In our single-centre, real-world retrospective study evaluating patients with advanced melanoma, the frequency of HPD was 15.2%. This rate is higher than those reported in previous studies, likely due to differences in the definitions of HPD used. HPD was significantly associated with an ECOG performance status ≥2, elevated LDH levels, liver metastasis, three or more metastatic sites, and an AEC < 100/μL. Additionally, the MDA-ICI and RMH scores were identified as useful tools for predicting HPD, which could be integrated into clinical practice to stratify patients’ risk of rapid progression.

Identifying predictive factors for HPD before initiating ICI therapy is crucial for determining which patients require close monitoring and early radiological assessment. Early identification and monitoring of high-risk patients may improve outcomes by allowing timely intervention and optimising treatment decisions. Patients identified as high risk may benefit from more personalised management approaches, including more frequent follow-up or alternative therapeutic strategies. Further studies are needed to validate these findings and to explore which therapeutic approaches may be more effective in managing high-risk patients for HPD, as the optimal treatment strategies for this group remain unclear.

## Figures and Tables

**Table 1:** Comparison of variables between HPD and non-HPD Groups.

Variable	HPD (*n* = 24)	Non-HPD (*n* = 134)	*p*-Value
Median age (year, range)	53.3 (37.1–75.1)	58.2 (21.6–80.4)	0.300
Age (y), *n* (%)			0.052
≥65	3 (12.5)	43 (32.1)	
<65	21 (87.5)	91 (67.9)	
Sex, *n* (%)			0.120
Female	13 (54.2)	50 (37.3)	
Male	11 (45.8)	84 (62.7)	
BMI, mean (kg/m^2^, range)	23.4 (14.9–35.6)	25.2 (16.4–43.0)	0.707
Histologic subtype, *n* (%)			0.740
Acral	4 (16.7)	24 (17.9)	
Nonacral cutenous	8 (33.3)	50 (37.3)	
Mucosal	3 (12.5)	8 (6.0)	
Uveal	2 (8.3)	6 (4.5)	
Unknown	7 (29.2)	46 (34.3)	
BRAF status, *n* (%)			0.843
Mutant	9 (37.5)	44 (32.8)	
Wild	15 (62.5)	89 (66.4)	
Unknown	0 (0.0)	1 (0.7)	
Metastatic sites, *n* (%)			
Liver	12 (50.0)	27 (20.1)	0.002
Lung	15 (62.5)	63 (47.0)	0.162
Bone	11 (45.8)	40 (29.9)	0.123
Brain	4 (16.7)	16 (11.9)	0.741
Number of metastatic sites, *n* (%)			<0.001
<3	6 (25.0)	86 (64.2)	
≥3	18 (75.0)	48 (35.8)	
Types of ICI, *n* (%)			
PD-1 inhibitor	16 (66.7)	89 (66.4)	0.981
PD-1-CTLA-4 combination	8 (33.3)	45 (33.6)	
Previous treatments *n* (%)			
BRAF+MEK inhibitors	6 (25.0)	24 (17.9)	0.572
Chemotherapy	6 (25.0)	20 (14.9)	0.236
Previous ICI *n* (%)			
CTLA-4 inhibitor	5 (20.8)	23 (17.2)	0.840
PD-1 inhibitor	4 (19.0)	17 (12.7)	
No	15 (62.5)	94 (70.1)	
Line of treatment *n* (%)			
1	10 (41.7)	79 (59.0)	0.116
≥2	14 (58.3)	55 (41.0)	
ECOG performance status *n* (%)			
0–1	18 (75.0)	125 (93.3)	0.013
2–4	6 (25.0)	9 (6.7)	
LDH, *n* (%)			
Normal	6 (30.0)	71 (56.3)	0.004
>ULN	5 (25.0)	37 (29.4)	
>1.5xULN	9 (45.0)	18 (14.3)	
Albumin, g/dl, *n* (%)			
<4.0	5 (26.3)	39 (31.2)	0.667
≥4.0	14 (73.7)	86 (68.8)	
CRP, mg/dl, *n* (%)			
≤0.5	6 (37.5)	53 (50.5)	0.333
>0.5	10 (62.5)	52 (49.5)	
AEC/μL, *n* (%)			
<100	11 (57.9)	37 (28.7)	0.011
≥100	8 (42.1)	92 (71.3)	
NLR, *n* (%)			
≤5.0	14 (73.7)	110 (84.6)	0.320
>5.0	5 (26.3)	20 (15.4)	
PLR, *n* (%)			
≤200	9 (47.4)	90 (69.2)	0.059
>200	10 (52.6)	40 (30.8)	
LMR, *n* (%)			
>2.78	12 (63.2)	64 (49.2)	0.257
≤2.78	7 (36.8)	66 (50.8)	
MPV/lymphocyte, *n* (%)			
>6.0	13 (68.4)	61 (46.9)	0.080
≤6.0	6 (31.6)	69 (53.1)	
GRIm score, *n* (%)	1.0 (0.0–3.0)	1.0 (0.0–3.0)	0.063
MDA-ICI score, *n* (%)			
Low risk	6 (31.6)	82 (64.6)	0.002
Intermediate risk	8 (42.1)	38 (29.9)	
High risk	5 (26.3)	7 (5.5)	
RMH score, *n* (%)			
Low risk	8 (40.0)	96 (76.8)	0.001
High risk	12 (60.0)	29 (23.2)	

Continuous variables are presented as median (range), while categorical variables are presented as *n* (%). Data for some variables were not available for all patients; therefore, the number of patients (*n*) reported for each variable reflects the available data. Abbreviations: BMI = Body Mass Index; ECOG = Eastern Cooperative Oncology Group; LDH = Lactate Dehydrogenase; CRP = C-Reactive Protein; AEC = Absolute Eosinophil Count; ULN = Upper Limit of Normal. A *p*-value of <0.05 was considered statistically significant.

**Table 2:** Summary of clinical variables in patients with HPD.

Patient	Age/Gender	Histologic Subtype	BRAF Status	Types of ICI	Liver Metastasis	Lung Metastasis	Brain Metastasis	Number of Metastatic Sites	ECOG	LDH U/L	AEC/μL	MDA-ICI Score	RMH Score
1	F	Unknown	Wild type	PD-1 inhibitor	No	Yes	No	3	2	152	170	Low risk	Low risk
2	F	Acral	Mutated	PD-1 inhibitor	No	No	Yes	3	0	605	50	Low risk	High risk
3	F	Nonacral cutaneous	Wild type	PD-1 inhibitor	Yes	Yes	No	5	2	365	20	Intermediate risk	High risk
4	F	Nonacral cutaneous	Mutated	PD-1 inhibitor	Yes	No	No	3	0	297	120	Intermediate risk	High risk
5	M	Mucosal	Wild type	PD-1 inhibitor	No	No	No	3	1	NA	NA	NA	NA
6	M	Unknown	Mutated	PD-1 inhibitor	Yes	Yes	No	3	1	NA	NA	NA	NA
7	F	Nonacral cutaneous	Mutated	PD-1 inhibitor	No	No	Yes	3	2	113	200	Intermediate risk	Low risk
8	M	Acral	Wild type	PD-1 inhibitor	No	No	No	2	0	515	10	Intermediate risk	Low risk
9	M	Acral	Wild type	PD-1 inhibitor	No	Yes	No	3	1	303	210	Low risk	High risk
10	M	Unknown	Wild type	PD-1 inhibitor	No	Yes	No	5	1	230	190	Low risk	High risk
11	M	Nonacral cutaneous	Wild type	PD-1 inhibitor	Yes	Yes	Yes	7	1	431	10	High risk	High risk
12	M	Nonacral cutaneous	Mutated	PD-1 inhibitor	No	No	No	3	2	1228	40	High risk	High risk
13	F	Unknown	Mutated	PD-1 inhibitor	No	No	No	1	0	120	50	Intermediate risk	Low risk
14	F	Mucosal	Wild type	PD-1 inhibitor	No	Yes	No	2	0	167	50	Low risk	Low risk
15	F	Uveal	Wild type	PD-1 inhibitor	Yes	Yes	No	3	0	3639	90	High risk	High risk
16	M	Mucosal	Wild type	PD-1 inhibitor	Yes	Yes	No	3	1	NA	NA	NA	NA
17	F	Acral	Wild type	PD-1-CTLA-4 combination	Yes	No	No	4	1	482	90	Intermediate risk	High risk
18	F	Unknown	Wild type	PD-1-CTLA-4 combination	No	No	No	2	0	NA	NA	NA	NA
19	F	Nonacral cutaneous	Mutated	PD-1-CTLA-4 combination	No	Yes	Yes	2	0	336	90	Low risk	Low risk
20	M	Uveal	Wild type	PD-1-CTLA-4 combination	Yes	Yes	No	3	0	1360	NA	NA	High risk
21	M	Nonacral cutaneous	Mutated	PD-1-CTLA-4 combination	Yes	Yes	No	6	2	273	290	High risk	High risk
22	F	Nonacral cutaneous	Wild type	PD-1-CTLA-4 combination	Yes	Yes	No	2	0	222	120	Intermediate risk	Low risk
23	F	Unknown	Wild type	PD-1-CTLA-4 combination	Yes	Yes	No	4	1	1384	80	Intermediate risk	High risk
24	M	Unknown	Mutated	PD-1-CTLA-4 combination	Yes	Yes	No	4	2	200	640	High risk	Low risk

Abbreviations: ICI, immune checkpoint inhibitor; ECOG, Eastern Cooperative Oncology Group; LDH, lactate dehydrogenase; AEC, absolute eosinophil count; MDA-ICI, MD Anderson immune checkpoint inhibitor score; RMH, Royal Marsden Hospital score; NA, not available.

**Table 3:** Multivariate analysis of variables associated with HPD.

	Model-1		Model-2 (MDA-ICI Score)		Model-3 (RMH Score)	
Variable	HR (95% CI)	*p*	HR (95% CI)	*p*	HR (95% CI)	*p*
Age (y)						
<65	Reference	0.168			Reference	0.114
≥65	0.315 (0.061–1.631)				0.259 (0.048–1.383)	
ECOG performance status						
0–1	Reference	0.056			Reference	0.023
2–4	3.761 (0.966–14.640)				4.523 (1.227–16.676)	
Number of metastatic sites						
<3	Reference	0.120	Reference	0.035		
≥3	3.007 (0.750–12.059)		3.546 (1.093–11.507)			
Liver metastasis						
No	Reference	0.894			Reference	0.555
Yes	1.093 (0.298–4.012)				1.426 (0.439–4.633)	
LDH						
Normal	Reference					
>ULN	1.677 (0.433–6.497)	0.455				
≥1.5 ULN	2.522 (0.602–10.556)	0.205				
AEC/μL						
<100	2.332 (0.713–7.626)	0.161	2.960 (1.029–8.511)	0.044	2.461 (0.814–7.446)	0.111
≥100	Reference		Reference		Reference	
RMH score						
Low risk					Reference	0.026
High risk					3.675 (1.166–11.580)	
MDA-ICI score						
Low risk			Reference			
İntermediate risk			2.375 (0.736–7.670)	0.148		
High risk			4.466 (0.947–21.061)	0.059		

Hazard ratios (HR) are presented with 95% confidence intervals (CI). Variables included in the multivariate analysis were selected based on their significance in the univariate analysis. Abbreviations: ECOG = Eastern Cooperative Oncology Group; LDH = Lactate Dehydrogenase; AEC = Absolute Eosinophil Count; ULN = Upper Limit of Normal; ICI = Immune Checkpoint Inhibitor; MDA-ICI = MD Anderson Immune Checkpoint Inhibitor Score; RMH = Royal Marsden Hospital Score. A *p*-value of <0.05 was considered statistically significant.

## Data Availability

The datasets used and/or analysed during the current study are available from the corresponding author on reasonable request.

## Supplementary Materials

The following supporting information can be downloaded at: https://www.mdpi.com/article/10.3390/curroncol31100472/s1, Table S1: Criteria for HPD Diagnosis and Their Frequency in HPD Patients. Table S2: ROC Analysis Results.
