# Supplementary material for: Exploring the Frequency and Risk Factors of Hyperprogressive Disease in Patients with Advanced Melanoma Treated with Immune Checkpoint Inhibitors
Source: Curr Oncol. 2024 Oct 18;31(10):6343–55. doi: 10.3390/curroncol31100472 (PMC11505979; doi:10.3390/curroncol31100472)
Supplement: Supplementary file 1 [file curroncol-31-00472-s001.zip › curroncol-3223801-supplementary.pdf]

Table S1: Criteria for HPD Diagnosis and Their Frequency in HPD Patients	
TTF of less than 2 months,	14 (53.8)
An increase of ≥ 50% in the sum of target lesions between baseline and the first radiologic evaluation,	13 (50.0)
The appearance of at least two new lesions in an organ already involved between baseline and the first radiologic evaluation	18 (69.2)
The spread of disease to a new organ between baseline and the first radiologic evaluation	22 (84.6)
Clinical deterioration with a decrease in ECOG performance status by ≥2 during the first 2 months	22 (84.6)

The data presented reflect the number and percentage of patients who met each HPD criterion, Abbreviations: ECOG = Eastern Cooperative Oncology Group. HPD= Hyperprogressive disease, TTF = Time-to-Treatment Failure

Table S2: ROC Analysis Results						
	AUC	95%CI	P value	Cut-off value	Sensitivity	Specificity
SII	0,582	0,427-0,737	0,250	-	-	-
PIV	0,568	0,412-0,724	0,339	-	-	-
SIRI	0,516	0,367-0,665	0,820	-	-	-
HALP	0,615	0,474-0,756	0,106	-	-	-
GRIm	0,631	0,497-0,764	0,066	-	-	-

AUC values indicate the discriminatory power of each variable in predicting HPD, with an AUC of 1.0 representing perfect discrimination and 0.5 indicating no discrimination. The cut-off values, along with their corresponding sensitivity and specificity, were determined to optimize the prediction of HPD. A p-value of <0.05 was considered statistically significant. Abbreviations**:** AUC = Area Under the Curve; CI = Confidence Interval; SII = Systemic Immune-Inflammation Index; PIV = Pan-Immune-Inflammation Value; SIRI = Systemic Inflammation Response Index; HALP = Hemoglobin, Albumin, Lymphocyte, and Platelet; GRIm = Gustave Roussy Immune
